# Editorial: Current trends in food processing and nutrition to mitigate nutritional health issues

**DOI:** 10.3389/fnut.2023.1278469

**Published:** 2023-09-12

**Authors:** Rana Muhammad Aadil, Monica Trif, Seydi Yıkmış, Muhammad Shahid Riaz Rajoka

**Affiliations:** ^1^National Institute of Food Science and Technology, University of Agriculture, Faisalabad, Pakistan; ^2^Department of Food Research, Centre for Innovative Process Engineering (Centiv) GmbH, Syke, Germany; ^3^Nutrition and Dietetics, Institute of Health Sciences, Tekirdağ Namık Kemal University, Tekirdağ, Türkiye; ^4^School of Dentistry, University of Maryland, Baltimore, Baltimore, MD, United States

**Keywords:** food processing, fortification, emerging technique, phytochemicals, bioactive compounds

This Research Topic was created to promote new studies exploring novel techniques for improving modern dietary choices. innovative approaches to reducing nutrient losses in present methods of food preparation, processing, preservation, extraction, and utilization; integration of functional foods rich in particular bioactive substances, and micro/macro-nutrients with anti-hyperlipidemic, anti-diabetic, anti-inflammatory, anti-carcinogenic, potentials, and so on, are among top priority goals set forward for achieving protected, healthy, and sustainable living. Ten articles (5 review articles and 5 original articles) are published in this Research Topic and a short summary of all these published articles is discussed below:

In a research article, the authors aimed to evaluate the anti-diabetic potential of *Abelmoschus esculentus* (okra) seed extract. Microwave-assisted extraction (MAE) was used to find the best parameters for extracting polyphenolic chemicals from okra seeds. For optimization, a face-center composite design was adopted. The polyphenolic content was investigated while the solvent/dry matter ratio, wavelength, and time were taken into account. Thin layer chromatography (TLC) and Fourier transform infrared (FTIR) spectroscopy were used to characterize the extract obtained under ideal conditions, and it was subsequently examined for anti-oxidant, alpha-amylase inhibitory, and anti-diabetic activity. The best conditions for phenols extraction were determined using response surface methodology (RSM) as microwave power 330 W and a solvent ratio (97.04/1 mL/g) for extraction time of 9.5 min. TLC found that the extract included quercetin and catechin and had a phenolic concentration (86.37 ± 1.13 mg GAE/g). Extract displayed good *in vitro* antioxidant capabilities with 2,2-diphenyl-1-picrylhydrazyl (DPPH) activity and FRAP assay, and functional groups indicative of polyphenols was found on FTIR spectra. The DPPH scavenging test yielded an IC_50_ of 3.99 ± 0.15 g/mL. In an α-amylase inhibitory assay, the optimized okra extract performed as a non-competitive inhibitor of pig pancreatic amylase (IC_50_ of 484.172.33 g/mL). The extract exhibited anti-diabetic efficacy in streptozotocin-induced diabetic male Wistar rats, as measured by food intake, serum lipid profile, fasting blood glucose levels, and changes in body weight, among other things (Woumbo et al.).

A review was conducted to evaluate the parameters impacting sweet potato (SP)'s moisture loss, drying kinetics, serum lipid profile, pre-treatments, conditions for operation, various drying methods, and their efficacy to enhance the functional, and nutritional properties as well as drying process. To acquire concentrated nutrients to enhance energy efficiency while being environmentally friendly, an optimal drying procedure is required. Traditional ways of drying SPs, such as sun or open-air drying stated as long process, which resulted the lower quality. So, different drying methods, such as freeze, infrared and vacuum drying, as well as pre-treatments such as ultrasound and osmotic dehydration, which were widely utilized around the world. The best-fit thin-layer models (Hii, Page, two-term, logarithmic) used for drying SP are also discussed, as are acceptable modeling methodologies for optimizing drying procedures (Rashid et al.).

Another review paper covered bisphenol A (BPA), which is a synthetic chemical that was commonly used in the synthesis of polycarbonate plastics, epoxy resins and polymer materials. BPA is present in the environment, for example, in food containers, toys, medical gadgets, thermal papers, drink bottles, and so on, and is leached into soil/water. It has the ability to change various biological systems because it is a powerful endocrine disruptor. Studies have verified its anti- androgen action and estrogen-like actions, which have a wide range of detrimental health effects, particularly on the neuroendocrine function, immunological system, and reproductive mechanism. According to recent scientific studies, it can also cause mutagenesis and carcinogenesis. The authors concentrated on the presence and amounts of BPA in various settings and dietary sources, as well as the fundamental processes of BPA- induced toxicity and health disturbances. It is a one-of-a-kind review in that it focuses on the link between BPA and cancer, hormone disturbance, and infertility immunosuppression. These problems are widespread today, and BPA plays a substantial role in their occurrence due to its extensive use in everyday utensils and other accessories. The study also examines research-based mitigation strategies for the harmful chemical (Manzoor et al.).

The authors' goal in this study is to review the accomplishments of pulsed electric field (PEF) used to the aging of fermented wine in a systematic manner. According to research on the use of PEF treatment, and PEF in a fermented wine provides the following benefits: (1) reducing the time it takes for brewing materials to macerate; (2) facilitating the extraction process of primary functional compounds; (3) improving the color of fermented wine; (4) deactivating spoilage bacteria; and (5) accelerating the synthesis of scent substances. These are mostly related to changes in molecular structure, PEF-induced bio-membrane electroporation, and the occurrence of chemical reactions. Furthermore, the essential features of PEF treatments after wine fermentation were highlighted, as well as some undesirable effects and future research prospects (Feng et al.).

The authors worked on the potential anti- aging impact of sea cucumber peptides (SCPs) on Caenorhabditis worm model as well as the underlying mechanisms in this study, which used SCPs from *Acaudina leucoprocta*, which were produced using patented bio-enzyme digesting method. SCPs increase the average nematode lifespan by 31.46%. SCPs improve *C. elegans*' anti- stress capacity by boosting motility and heat resistance. Additionally, collected oxidative stress inducers including the reduction of ROS (71.43%) and lipofuscin (40.84%). Furthermore, SCPs can boost nematode antioxidant capacity by increasing SOD and CAT activity and decreasing MDA buildup in nematodes by 32.44% (Wu et al.).

The increasing variety of food processing approaches the ongoing expansion of the food-trade chain, and possibility of threat factors in the process of making food all make people pay a growing amount of devotion to the facility creation, and enhancement of the Hazard Analysis Critical Control Point (HACCP) system. Only endpoint control and afterwards monitoring of food can provide ultimate food safety. It is critical to strictly detect and analyze food safety issues during the processing process. There is a need for the manufacturing companies to establish and implement the HACCP systems, so it is necessary to carry out the primary responsibility of food safety as well as improve the practical application and theoretical understanding of HACCP system in China. The study's objectives were to track the trends and influence of research in this field by Chinese research institutions and significant authors, and to analyze the research. It is crucial for HACCP research to continue. The study's findings revealed that (1) there was a steady increase in HACCP publications in China from 1992 to 2004 before they started to decline; (2) the indexes of journals with more publications were more concentrated, with Food Science publishing the most; and (3) the cultivation bases of the State Key Laboratory of Chinese Medicinal Materials in the Center of Chinese Medicine Resources of China were among the largest research institutions; (4) four additional active research teams have been established in the field of HACCP as a result of the main author indicators. To ensure that food is truly safe, it is advised that China integrate food hazard analysis and assessment into the pre and post-production processes of food (Shi et al.).

In a review study, authors described how residual agro-waste from the pomegranate juice, which was valorized to make pomegranate seed oil (PSO) as well as sunflower oilseed cake was utilized to manufacture the sunflower meal protein concentrate (SMPC). After being removed, both components were mixed to make high nutria omega cookies. A thorough set of laboratory analytical techniques were used to assess the properties of pomegranate seed oil in order to verify its purity and viability. Then, using different concentrations of PSO and SMPC, the HNO5 cookie products were made. The sensory, proximate, physicochemical, and efficacy tests of these cookies were thoroughly examined in order to ascertain their general shelf-life properties and nutritional qualities. The findings showed that 15% SMPC and 15% PSO cookies exhibited stability in a number of sensory tests and physicochemical. Rats' responses to famine were dramatically reduced by the punicic acid in HNO5 cookies, which also gradually enhanced various metabolic functions and general health profiles (Iqbal et al.).

In a trial, the formulated baby meal was made from *Eleusine coracana* (ragi) and *Musa paradisiaca* (Nendran banana). Formulated weaning food was examined using a variety of established techniques, proving that it could give growing infants the nutrients they needed for healthy growth and development. Weaning food's ability to last for 3 months in two different packaging types of aluminum and plastic was also investigated. The aluminum foil pouch showed the best ability to last. This ready-to-serve food could be viewed as a highly effective supplemental food for infants because it is made with natural ingredients that contain important macronutrients and micronutrients. Additionally, this breakthrough may lead to the introduction of a weaning solution that is specifically designed for poor socioeconomic groups (Kabeer et al.).

The various features of summer savory, including its medicinal qualities, biological activity, food applications, nutritional value, potential health advantages, and use as an addition in grill feed, were covered by the writers of a review study. Additionally, the associated toxicity is covered. Summer savory leaves are rich in total phenolic components, which have strong antioxidant effects. Summer savory contains rosmarinic acid as a primary ingredient. According to phytochemical studies, the main constituents of Satureja species include tannins, gums, phenolic compounds, mucilage, volatile oils, acids, sterols, pyrocatechol, and pyrocatechol. In tests for antioxidant, cytotoxic, and antibacterial effects, summer savory extract demonstrates significant biological potential. Summer savory extract exhibits an inhibitory effect on lipid peroxidation in terms of antioxidant activity. In addition to having minerals and vitamins, summer savory includes Fe (III) reductive and free radical scavenging properties. In addition to having antibacterial and antioxidant characteristics, summer savory also offers preventive effects against cancer, cardiovascular illnesses, Alzheimer's disease, Jurkat T cells, cholesterol, and infections. Due to their antioxidant qualities and high nutritional content, this plant's leaves and stems are used in the pharmaceutical industries and food feed. In conclusion, summer savory is usually regarded as being healthy for people because of its adaptable qualities and medicinal applications (Ejaz et al.).

Spray-dried yogurt powder (SDYP) offers functional qualities that enhance solubility, make other food derivatives such bread and pastries easier to use, process, package, and transport, and have shelf stability. The goal of the current study was to improve SDYP and expand its application in cookie preparation for functional purposes. Different outlet air temperatures (OAT) (65, 70, and 75 C) and input air temperatures (IAT) (150, 155, and 160 C) were used to spray-dry yogurt. However, *S. thermophilus* culture exhibits tolerance to the intense heat approaches, whereas spray drying demonstrates that increasing temperature and increases nutritional loss. The culture of *L. delbrueckii subsp*. Bulgaricus, on the other hand, was discovered to be considerably impacted. The growing concentration of SDYP, baking properties the protein profile and mineral of cookies were all examined for a direct proportional relationship. DPPH antioxidant activity, 2,2′-azino-bis (3-ethylbenzothiazoline-6-sulfonic acid), and total phenolic content were all considerably impacted. For all characteristics, the sensory profile shows a slope from T0 (0% SDYP) to T3 (10% SDYP), although it starts to slow down as the SDYP concentration rises over 15%. According to this study, a certain temperature combination (OAT: 60°C IAT: 150°C) may be utilized to maximize inoculation culture survival, and this powder can be used to create functional cookies with considerably better sensory and biochemical properties (P < 0.05) (Ali et al.).

## Author contributions

RA: Conceptualization, Writing—original draft, Writing—review and editing. MT: Writing—review and editing. SY: Writing—review and editing. MR: Writing—review and editing.

